# PDGFB-expressing mesenchymal stem cells improve human hematopoietic stem cell engraftment in immunodeficient mice

**DOI:** 10.1038/s41409-019-0766-z

**Published:** 2019-12-05

**Authors:** Xiuxiu Yin, Linping Hu, Yawen Zhang, Caiying Zhu, Hui Cheng, Xiaowei Xie, Ming Shi, Ping Zhu, Xueying Zhao, Wanqiu Chen, Lu Zhang, Cameron Arakaki, Sha Hao, Mei Wang, Wenbin Cao, Shihui Ma, Xiao-Bing Zhang, Tao Cheng

**Affiliations:** 1 State Key Laboratory of Experimental Hematology, National Clinical Research Center for Blood Diseases, Institute of Hematology & Blood Diseases Hospital, Chinese Academy of Medical Sciences & Peking Union Medical College, Tianjin, 300020 China; 2 Department of Medicine, Loma Linda University, 11234 Anderson Street MC1528B, Loma Linda, CA USA; 3 Center for Stem Cell Medicine, Department of Stem Cell & Regenerative Medicine, Chinese Academy of Medical Sciences & Peking Union Medical College, Tianjin, 300020 China

**Keywords:** Haematopoietic stem cells, Bone marrow transplantation

## Abstract

The bone marrow (BM) niche regulates multiple hematopoietic stem cell (HSC) processes. Clinical treatment for hematological malignancies by HSC transplantation often requires preconditioning via total body irradiation, which severely and irreversibly impairs the BM niche and HSC regeneration. Novel strategies are needed to enhance HSC regeneration in irradiated BM. We compared the effects of EGF, FGF2, and PDGFB on HSC regeneration using human mesenchymal stem cells (MSCs) that were transduced with these factors via lentiviral vectors. Among the above niche factors tested, MSCs transduced with PDGFB (PDGFB-MSCs) most significantly improved human HSC engraftment in immunodeficient mice. PDGFB-MSC-treated BM enhanced transplanted human HSC self-renewal in secondary transplantations more efficiently than GFP-transduced MSCs (GFP-MSCs). Gene set enrichment analysis showed increased antiapoptotic signaling in PDGFB-MSCs compared with GFP-MSCs. PDGFB-MSCs exhibited enhanced survival and expansion after transplantation, resulting in an enlarged humanized niche cell pool that provide a better humanized microenvironment to facilitate superior engraftment and proliferation of human hematopoietic cells. Our studies demonstrate the efficacy of PDGFB-MSCs in supporting human HSC engraftment.

## Introduction

Postnatal hematopoietic stem cells (HSCs) are mainly localized in specialized niches of the bone marrow (BM), and these niches are essential for maintaining the homeostasis of HSCs [1]. In HSCs transplantation, preconditioning via irradiation or chemotherapeutics is usually performed to ablate the host stem cells to allow donor HSCs implantation [2]. Without preconditioning, donor chimerism cannot be established or is very low [3]. However, preconditioning by irradiation not only depletes the recipient’s HSCs but also disrupts their niche function by damaging sinusoidal blood vessels and stromal cells [4, 5]. In a humanized mouse model, niche regeneration is a prerequisite for complete reconstitution of transplanted HSCs and hematopoiesis [6, 7]. The low level of human HSCs engraftment in immunocompromised mice is partly due to the lack of an appropriately humanized microenvironment to support human hematopoiesis after transplantation. Therefore, in this study, we aimed to improve human hematopoietic engraftment using humanized niche cells.

Mesenchymal stem cells (MSCs) play an important role in maintaining tissue homeostasis, and they are widely recognized as a necessary component of niches [8]. In mouse models, human MSCs engrafted in murine BM are capable of integration into the hematopoietic microenvironment, which contributes to the maintenance of hematopoietic subgroups and enhance human HSCs engraftment in mice, however, with limited success [7].

Here, we aimed to enhance the functionality of MSCs by overexpressing human *EGF, FGF2*, or *PDGFB*. These factors were chosen due to their positive effects on MSCs proliferation and niche recovery after irradiation. The administration of EGF accelerates the recovery of long-term HSCs and improves the survival of mice following radiation-induced myelosuppression [9]. In addition, FGF2 signaling may support both MSCs and HSCs. For instance, during stress, the FGF signaling cascade is robustly amplified, resulting in an increase in MSCs pool, followed by the restoration and expansion of HSCs [10]. Furthermore, we have previously demonstrated [11] that the transplanted mouse Sca1^+^ cells overexpressing human *PDGFB* stimulate mouse MSC proliferation and recruit it to the endosteum to form mineralized trabecular bone. PDGFB also promotes angiogenesis, indicating that PDGFB can potentially modify the BM niche [11]. However, all of the above studies were conducted in mouse models, and whether EGF, FGF2, or PDGFB can positively affect the humanized niche and human hematopoietic cell engraftment remains unclear.

Among the above factors tested, PDGFB exhibited the most significant efficacy. Our data showed that the overexpression of *PDGFB* promoted MSC proliferation. There were more PDGFB-MSCs than GFP-MSCs engrafted after injection into the mouse BM. Consequently, the PDGFB-MSC-humanized microenvironment significantly improved human hematopoietic cells engraftment and better maintained their self-renewal properties in immunodeficient mice. This finding may have applications in promoting niche reconstitution and in vivo HSC expansion.

## Materials and methods

### Human cord blood processing

Human cord blood samples were obtained from Tianjin Obstetric Central Hospital (Tianjin, China) according to the protocol approved by the Ethical Committee on Medical Research at the Institute of Hematology. All the researches were conducted in accordance with the Declaration of Helsinki and patient informed consent. CD34^+^ cell isolation was performed as previously described [12]. Briefly, mononuclear cells were isolated by Ficoll–Hypaque density gradient centrifugation followed by CD34^+^ cell enrichment using the CD34^+^ microbead kit (Miltenyi Biotec; 130-046-703).

### Xenotransplantation and detection of human engraftment

Female NOD-SCID or NOG mice, 6–8 weeks old, were irradiated at a dose of 250 cGy 24 h before transplantation. For the CD34^+^ cell and MSC cotransplantations in NOD-SCID mice, cells were suspended in a minimum volume of 10 µl of phosphate-buffered saline. Each mouse was anesthetized, the knee was flexed, and the cells were injected into the joint surface of the right tibia by 28-gauge needle. For limiting dilution analysis, CD34^+^ cells (2500, 5000, 10,000, and 20,000) together with engineered MSCs were injected into one tibia of each mouse. For NOG mice, we injected MSCs in both tibiae and then transplanted human CD34^+^ cells intravenously. For serial transplantations, 1 × 10^7^ whole BM cells obtained separately from each primary recipient were intravenously transplanted into secondary recipient that exposed to sublethal irradiation. At 12 weeks (for NOD-SCID) or 16 weeks (for NOG) after transplantation, cells were collected from the IT (injected tibia), Non-IT (including non-injected tibia, two femurs), and spleen. After centrifugation, cells were resuspended with 100 μL of staining buffer and labeled with antibodies at 4 °C for 30 min. Then the cells were washed with 1 mL of staining buffer and analyzed by flow cytometry. Antibodies used in this study were shown in Table S1. FACS analysis was performed using BD LSRII or FACS Canto II (BD). Flow data analysis was performed using FlowJo software.

### RNA extraction and real-time RT-PCR

RNA was prepared using a miniRNA kit (QIAGEN) with on-column DNA digestion (QIAGEN). Total RNA was subjected to reverse transcription and then qPCR using SYBR green on a LightCycler 480 (Roche). The primers used in this study were shown in Table S2.

### RNA-seq library preparation and data analysis

Total RNA was extracted using RNA isolation kits (EXIQON). RNA-seq libraries were constructed using the NEBNext Ultra^TM^ RNA Library Prep Kit (NEB, USA) and sequenced 150-bp paired-end on an Illumina HiSeq X10 platform. For the raw sequencing outputs, first, we removed the reads with low-quality bases and adaptor contaminants by in-house Perl scripts. Then, the clean reads were aligned with the hg38 build of the human genome using the Salmon software (version 0.8.2). Next, differentially expressed genes were determined with the DESeq2 program [13], using the following thresholds: log_2_ (fold-change) ≥ 1 or ≤−1 and *p* value < 0.05. Finally, we used the clusterProfiler program [14] and GSEA [15] to identify the enriched biological processes and pathways among the differentially expressed genes. Database: RNA-seq for single accession numbers (GEO: GSE113857). See https://www.ncbi.nlm.nih.gov/geo/query/acc.cgi?acc=GSE113857 for more information and a full list of supported databases.

### Statistical analysis

Data are presented as the mean ± SEM and were visualized using the Prism Version 7.0 (GraphPad). The significance of the differences between groups was determined using Student’s *t* test and the Mann–Whitney test. *p* values < 0.05 were considered statistically significant. *p < 0.05; **p = 0.01–0.001; ***p < 0.001 in comparison. LDA was performed using L-Calc software (Stem cell Technologies).

## Results

### PDGFB-MSCs improve human hematopoietic cell engraftment in NOD-SCID mice

MSCs [16] were transduced with human *EGF, FGF2,* or *PDGFB* by lentiviral transduction and GFP served as a control (Fig. S1A). The expression of transduced genes was confirmed by RT-qPCR and Western blot analysis (Fig. S1B, C). In vitro proliferation experiment indicated that MSCs transduced with human EGF, FGF2, or PDGFB had stronger proliferation ability than GFP-MSCs. Among them, *PDGFB* overexpression promoted MSC self-expansion most obviously (2.3-fold compared with GFP-MSCs at 9 days of culture, *n* = 3, *p* < 0.05; Fig. S1D).

**Fig. 1:**
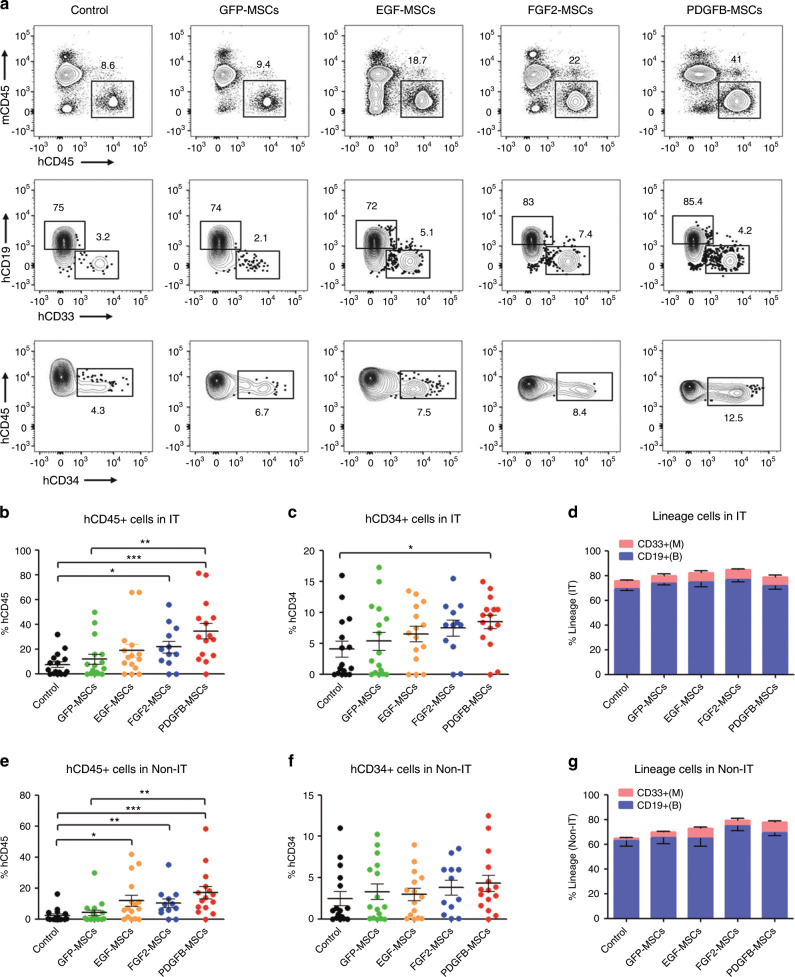
PDGFB-MSCs improve human hematopoietic cell engraftment in NOD-SCID mice. **a** Representative flow plots of human cell engraftment and multilineage differentiation 12-weeks post transplantation in the BM of NOD-SCID mice that were cotransplanted with human CB-CD34^+^ cells and MSCs over-expressing *GFP, EGF, FGF2*, or *PDGFB*. **b** Mean human cell engraftment in the injected tibia (IT). **c** The percentage of human CD45^+^CD34^+^ cells in the IT. **d** Lineage potential of human cells in the IT. **e **Mean human cell engraftment in the non-injected tibia (Non-IT) 12-weeks post transplantation. **f** The percentage of human CD45^+^CD34^+^ cells in non-IT. **g** Lineage potential of human cells in the Non-IT. *n* = 12–16 per group, *p < 0.05; **p = 0.01–0.001; ***p < 0.001.

The cotransplantation of MSCs facilitates the engraftment of hematopoietic cells [7]. In the prevent study, 2.6–3.0 × 10^4^ CB-CD34^+^ cells mixed with 5 × 10^5^ GFP-MSCs, EGF-MSCs, FGF2-MSCs or PDGFB-MSCs were injected into the right tibia. In the control group, only CB-CD34^+^ cells were injected. Overall engraftment and multilineage differentiation were assessed 12 weeks after transplantation. Previous studies [7, 17, 18] have shown that the cotransplantation of human MSCs and HSCs leads to accelerated hematopoietic recovery or increased chimerism (or both) in animal models and humans. In contrast, our study found that GFP-MSC only slightly increased the level of human engraftment compared to the control group in injected tibia (IT) (GFP-MSCs vs control: 11.8 ± 3.9% vs 7.5 ± 2.3%, *p* = 0.6; *n* = 12–16 per group, Fig. 1a, b). This discrepancy may be due to several reasons: (1) we reduced the number of transplanted MSCs: In our preliminary experiment, we cotransplanted human CD34^+^ cells with 1 × 10^6^ MSCs according to these previous studies. Similarly, the results showed a significant increase in the frequency of human CD45^+^ cells (Fig. S2A). However, considering the limited volume of transplanted cells caused by the limited tibia medullary cavity volume, we reduced the number of transplanted MSCs to 5 × 10^5^ according to a previous study [19]. Reducing the number of transplanted MSCs would reduce the effect of MSCs on the transplantation of human HSCs [20, 21]. Moreover, (2) the source of MSCs was different: MSCs used in our study were induced MSCs from human cord blood CD34^+^ cells by direct reprogramming [16]. MSCs involved in the previous studies were derived from bone marrow or lung. It has been demonstrated that different sources of MSCs may have different characteristics and functions [22, 23]. (3) The transplant site was different: in the cited articles, human HSCs were transplanted intravenously and had a homing effect. Cotransplantation of MSCs enhanced HSCs engraftment by improving the homing of HSCs as previously reported [24], which increased the difference between MSC group and non-MSC group. In our study, HSCs were injected directly into the tibia, which eliminated the homing effect, thereby attenuating the difference between MSC group and non-MSC group. Therefore, in our transplantation scheme, cotransplantation of 5 × 10^5^ GFP-MSCs showed only an upward trend in human cells engraftment, but no significant difference was observed. However, the cotransplantation of PDGFB-MSCs resulted in a significant increase (4.6-fold) in the frequency of human CD45^+^ cells compared with the non-MSC group (34.5 ± 6.2% vs 7.5 ± 2.3%, *p* < 0.001; Fig. 1a, b). Furthermore, the human engraftment levels were higher in PDGFB-MSC recipients than in GFP-MSC recipients (Fig. 1a, b). We also observed a significant increase in engraftment in the IT of FGF2-MSCs recipients compared with that in the control group (Fig. 1b). However, there was no difference compared with the GFP-MSC recipients (*p* > 0.05). There was no detectable difference between the EGF-MSC recipients and control group. The PDGFB-MSC recipients also had significantly more CD34^+^ cells than the control group (PDGFB-MSCs vs control: 8.5 ± 1.1% vs 4.1 ± 1.3%, *p* < 0.05; Fig. 1a, c). In all engrafted recipients, the majority of human cells in the IT were CD19^+^ B cells and CD33^+^ myeloid cells (Fig. 1a, d).

Then, we analyzed the noninjected bones to determine the migration and self-renewal capacity of the transplanted cells [25, 26]. We found that the overall engraftment levels in the noninjected tibia (Non-IT) mirrored the pattern in the injected bones, although the Non-IT engraftment was relatively lower than that in the injected bone (Fig. 1e–g). This analysis also demonstrated that PDGFB was the best factor to enhance human hematopoietic cell engraftment.

As was observed in the IT, the spleens (SP) of the PDGFB-MSC recipients had significantly more CD45^+^ cells than those of the control and GFP-MSC recipients (Fig. S2B). The majority of cells in the spleen were CD19^+^ B cells, including CD45^+^CD19^+^IgM^+^ mature B cells (Fig. S2C, D), suggesting that the PDGFB-MSCs did not affect the differentiation of the B cell lineage.

### PDGFB-MSCs support the self-renewal of long-term human HSCs

**Fig. 2:**
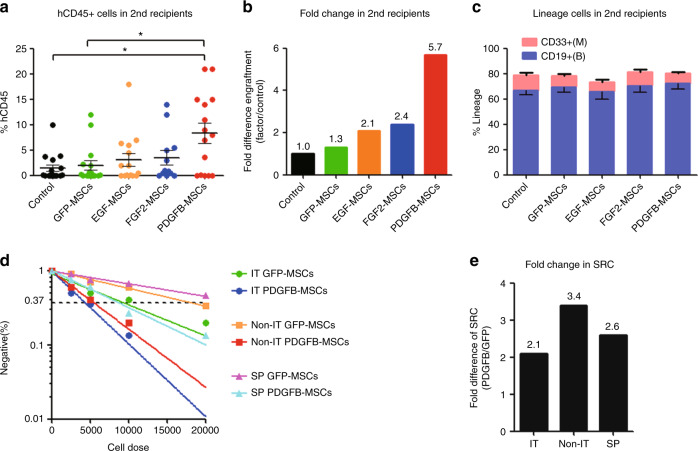
PDGFB-MSCs improve the SCID-repopulating cell (SRC) frequency in the IT, Non-IT, and SP, and support the self-renewal of human HSCs. **a** Mean human cell engraftment of secondary mice from each group. **b** Bars represent the fold difference of engraftment levels in secondary mice between cotransplanted MSCs and control group. **c** Lineage potential of human cells from secondary mice. **d** Frequency of human cells in NOD-SCID mice cotransplanted with GFP-MSCs and PDGFB-MSCs measured by limiting dilution analysis. **e** Bars represent fold increases in SRC frequency in PDGFB-MSCs cotransplanted recipients compared with GFP-MSCs in the IT, Non-IT, and SP, separately. *p < 0.05.

To determine whether engineered MSCs supported the engraftment and in vivo expansion of long-term HSCs, we performed secondary transplantation. Consistent with the previous reports by others [27] and our group [12], secondary recipients showed considerably lower levels of engraftment than the primary recipients (Fig. 2a). However, human cells derived from the primary PDGFB-MSCs recipient showed a 5.7-fold increase in secondary engraftment over the control group 12 weeks after transplantation (Fig. 2b). There was no significant difference in lineage differentiation of the engrafted human cells in the secondary recipients (Fig. 2c). All the results suggest that cotransplantation with PDGFB-MSCs promotes the self-renewal of human HSCs in primary recipients, leading to superior engraftment in secondary transplantation.

To quantitate human HSC engraftment, we conducted a functional SCID repopulation cell (SRC) assay. We performed limiting dilution analysis (LDA) with 2500, 5000, 10,000, and 20,000 CB-CD34^+^ cells and calculated the SRC frequency using L-Calc software [12]. The SRC frequency in the IT of PDGFB-MSC recipients was ~2.1-fold higher than that in the GFP-MSC recipients (1 in 4353 vs 1 in 8908; Fig. 2d, e; Table S3). Similar repopulation increases were also detected in the Non-IT and spleen (Fig. 2d, e; Table S3). These data demonstrate that the PDGFB-MSC-treated BM microenvironment is more effective at supporting human HSC reconstitution.

### PDGFB-MSCs improve human hematopoietic cell engraftment in NOG mice

**Fig. 3:**
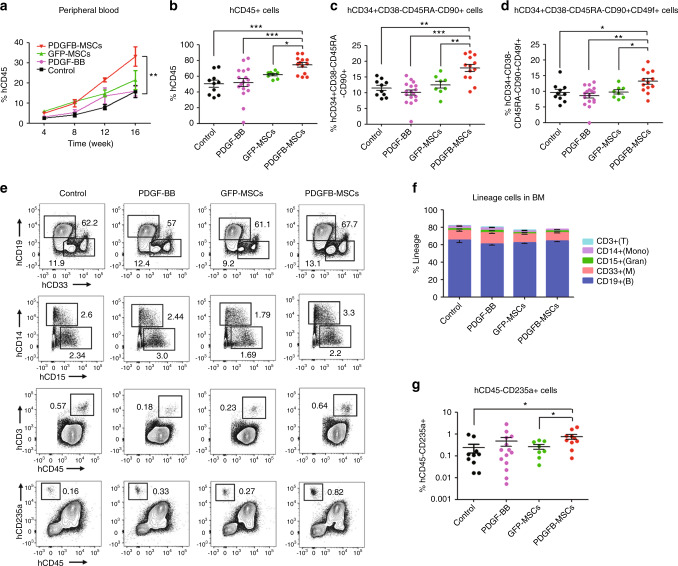
PDGFB-MSCs improve human hematopoietic cell engraftment in NOG mice. **a** Human cell engraftment in peripheral blood post transplantation. **b** Mean human CD45^+^ cells in the BM of NOG mice. **c** Level of BM engraftment in the primitive stem cell subsets: CD34^+^CD38^−^CD45RA^−^CD90^+^; and **d** CD34^+^CD38^-^CD45RA^−^CD90^+^CD49f^+^. **e** Representative FACS profiles showing multilineage repopulation of NOG mice in each group (CD19^+^ B cells, CD33^+^ myeloid cells, CD14^+^ monocytes, CD15^+^CD14^−^ granulocytes, CD3^+^ T cells and CD45^−^CD235a^+^ erythroid cells). **f** Lineage engraftment expressed as frequency of human CD45^+^ cells. **g** The level of human CD45^−^CD235a^+^ erythroid cells in each group. *n* = 8 −17 per group, three independent experiments; *p < 0.05; **p = 0.01 −0.001; ***p < 0.001.

To prevent artifacts of higher engraftment when using a certain immunodeficient mouse strain, we examined the engraftment in NOG mice, which are more receptive to human HSC implantation than NOD-SCID mice. In this research, we injected 5 × 10^5^ PDGFB-MSCs into two tibias of the NOG mice, followed by the intravenous transplantation of 0.2–1 × 10^5^ human CB-CD34^+^ cells. In addition, to determine whether inject of human PDGF-BB (PDGFB homodimeric protein) could mimic the effect of PDGFB-MSCs, we added human PDGF-BB treated group (0.5 mg/kg intravenously every 12 h for 2 days). At 16 weeks after transplantation, human CD45^+^ cells engraftment was significantly higher in the peripheral blood and BM of PDGFB-MSC-injected mice than that of the control, PDGF-BB and GFP-MSC groups (Fig. 3a, b). Importantly, larger proportions of the phenotypically defined human HSC cell subsets (CD90^+^ and CD49f^+^ HSCs subsets) [28] were observed within the PDGFB-MSC-treated mice than in the other three groups (Fig. 3c, d). However, human PDGF-BB treatment did not increase total human engraftment (Fig. 3a–d).

In all groups, multilineage differentiation was similar for CD19^+^ B cells, CD33^+^ myeloid cells, CD14^+^ monocytes, CD15^+^CD14^−^ granulocytes and CD3^+^ T cells (Fig. 3e, f). The PDGFB-MSC-treated mice produced a superior differentiation of CD45^−^CD235a^+^ erythroid cells to those of the control and GFP-MSC mice (PDGFB-MSCs vs control: 0.76 ± 0.2% vs 0.24 ± 0.1%, *p* < 0.05; PDGFB-MSCs vs GFP-MSCs: 0.76 ± 0.2% vs 0.26 ± 0.1%, *p* < 0.05; Fig. 3e, g). These results suggest that PDGFB-MSCs do not affect overall differentiation but favor the differentiation of HSCs into the erythroid lineage.

We found the same differential engraftment in the spleen as observed in the BM. The spleens of the PDGFB-MSC-treated mice had more human CD45^+^ cells than those of the control, PDGF-BB and GFP-MSC mice (Fig. S3A, B). Lineage analysis showed that the majority of cells in the spleens were CD19^+^ B cells, including CD19^+^IgM^+^ mature B cells. CD3^+^ T and CD56^+^ NK cells were also detected in all spleens, albeit at lower levels (Fig. S3A, C).

To quantitate long-term HSCs, we conducted secondary transplantations in NOG mice. Similar to the results obtained in the NOD-SCID mice, the presence of functional, self-renewing HSCs within the PDGFB-MSC-treated mice was confirmed by higher engraftment in secondary recipients (PDGFB-MSCs vs control: 10.6 ± 2.4% vs 3.2 ± 1.3%, *p* < 0.05, Fig. S4). These data demonstrate that the niche that is humanized by PDGFB-MSC implantation supports the self-renewal and proliferation of human primitive HSCs in NOG mice.

### PDGFB-MSCs have a distinct gene expression signature

**Fig. 4:**
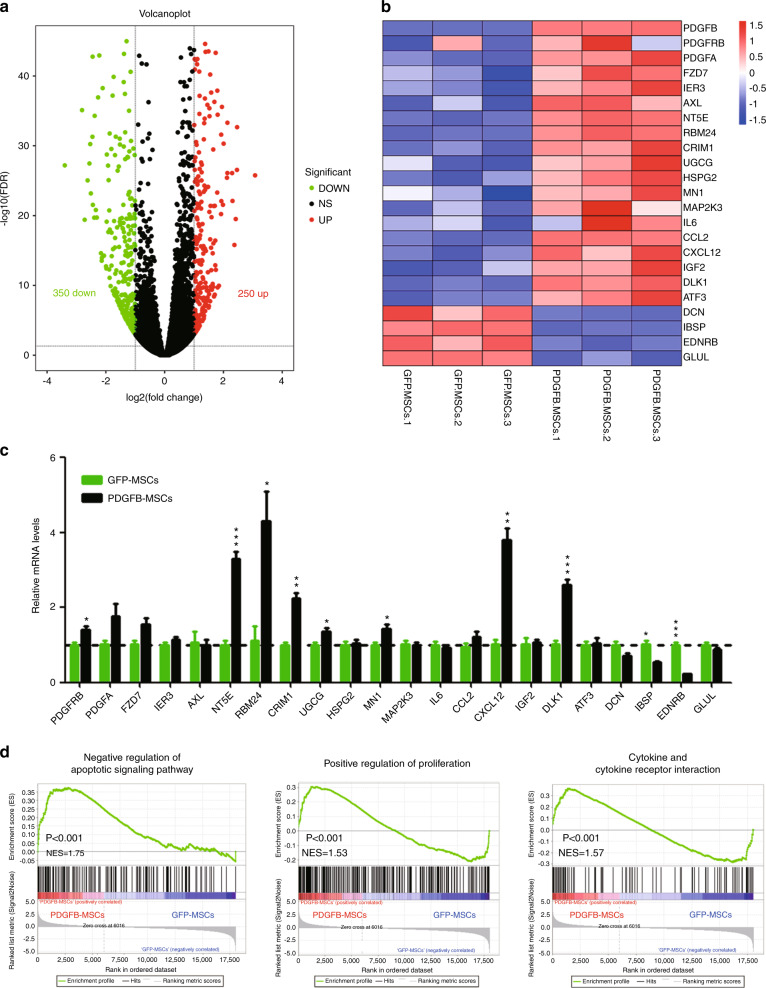
PDGFB-MSCs have a distinct gene expression signature. **a** Scatter plot of global mRNA profiling showing upregulated (red) and downregulated (green) genes in PDGFB-MSCs versus GFP-MSCs (false discovery rate (FDR) ≤ 0.05, |fold-change| ≥ 2). A total of 600 differentially expressed genes (250 up- and 350 downregulated) were identified between the PDGFB-MSCs and GFP-MSCs. **b** Heatmap of MSC autocrine/paracrine genes, stemness-related genes, and differentiation genes differentially expressed between PDGFB-MSCs and GFP-MSCs. **c** Real-time qPCR analysis of related genes in the PDGFB-MSCs and GFP-MSCs. **d** Gene set enrichment analysis of the negative regulation of apoptotic signaling, positive regulation of proliferation and cytokine and cytokine receptor interaction in PDGFB-MSCs and GFP-MSCs. *p < 0.05; **p = 0.01–0.001; ***p < 0.001.

To elucidate the molecular mechanism of PDGFB-MSCs promoting human engraftment, we performed RNA sequencing of cultured GFP-MSCs and PDGFB-MSCs. A total of 600 differentially expressed genes (250 upregulated and 350 downregulated) were identified between the PDGFB-MSCs and GFP-MSCs (Fig. 4a). As expected, *PDGFB* was the most highly expressed gene in the PDGFB-MSCs compared with the GFP-MSCs (Fig. 4b). *PDGFB* and *PDGFRB*, whose transcripts were associated with the MSC engraftment and regenerative capacity in vivo through the PDGFB signal, were upregulated in the PDGFB-MSCs. Genes indicative of MSC stemness (*NT5E, RBM24, CRIM1, UGCG, MN1*) [29] were upregulated and differentiation genes (*IBSP, EDNRB*) [29] were downregulated in PDGFB-MSCs compared to GFP-MSCs. Furthermore, *CXCL12* [30–33] and *DLK1* [34] upregulated in PDGFB-MSCs were implicated in the maintenance of human HSCs (Fig. 4b, c). These data indicate that PDGFB-MSCs undergo well-controlled proliferation and differentiation in vitro. Gene set enrichment analysis revealed that the negative regulation of apoptotic signaling, positive regulation of proliferation and cytokine and cytokine receptor interaction were enriched in the PDGFB-MSCs compared to GFP-MSCs (Fig. 4d), which indicates that PDGFB-MSCs have the ability to undergo less apoptosis and proliferate faster than GFP-MSCs. These characteristics may become more obvious after transplantation into the irradiated mouse BM. Taken together, these analyses indicate that PDGFB-MSCs resembled stromal progenitors, which could better provide a supply of human niche factors for the humanized microenvironment and would be sufficient to promote superior implantation and growth of human hematopoietic cells.

### Better survival and proliferation of PDGFB-MSCs than GFP-MSCs in the bone marrow cavity

**Fig. 5:**
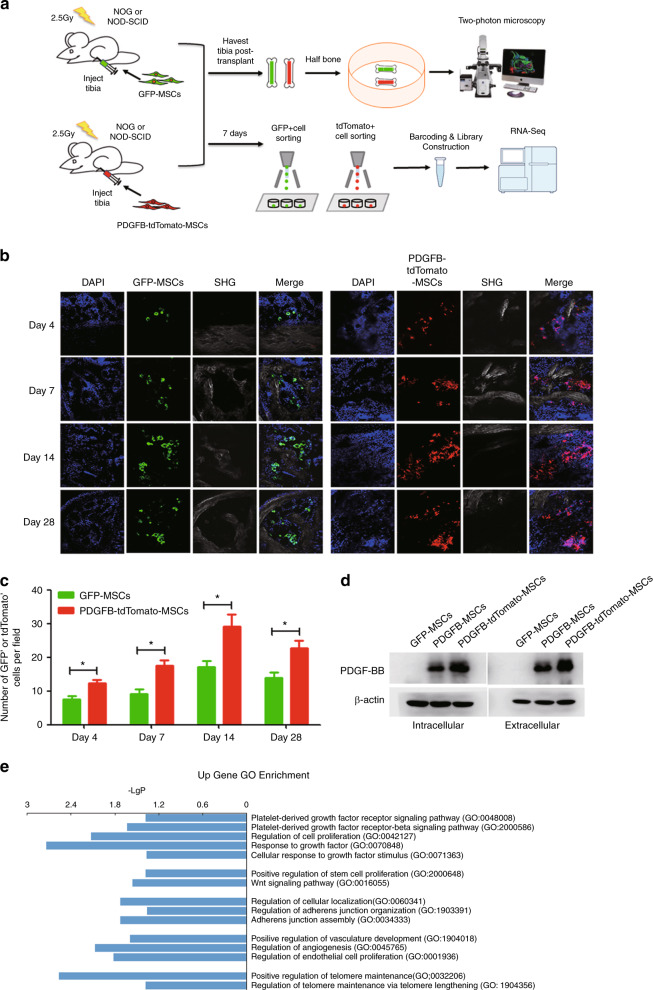
PDGFB-MSCs proliferate more than GFP-MSCs in bone marrow cavity. **a** Schematic representation of the experimental design. **b** Tracing of GFP^+^ and tdTomato^+^ cells by two-photon fluorescence microscopy at different time points after transplantation in the bone marrow cavity. **c** Quantification of the number of GFP^+^ and tdTomato^+^ cells per field in the injected tibias at multiple time points after transplantation. **d** Western blot of intracellular and extracellular PDGFB protein levels in GFP-MSCs, PDGFB-MSCs, and PDGFB-tdTomato-MSCs. **e** GO enrichment analysis of upregulated genes in PDGFB-tdTomato-MSCs harvested 1 week after transplantation (*p* < 0.05). SHG, Second harmonic generation. *p < 0.05.

We further investigated the potential mechanisms of the PDGFB-MSC-mediated in vivo long-term engraftment of human HSCs. MSCs have been reported in numerous studies to enhance HSC engraftment. However, the long-term survival and proliferation of transplanted MSCs in host animals either by intravenous injection or intrabone injection have not been convincingly demonstrated. Nevertheless, we believe that the short-term survival and proliferation of MSCs are critical to convey the positive effects on HSC survival, self-renewal, and expansion. Therefore, we hypothesized that the overexpression of *PDGFB* promotes MSC expansion. In support of this notion, *PDGFB* overexpression promoted MSC self-expansion in vitro (Fig. S1D). To investigate whether *PDGFB* overexpression endows MSCs with better survival and a proliferative advantage, we transplanted 5 × 10^5^ GFP-MSCs or PDGFB-tdTomato-MSCs (we engineered the PDGFB-MSCs to express tdTomato fluorescent protein by lentiviral transfection) into the tibias of irradiated immunodeficient mice (Fig. 5a). The tibias from transplanted mice were isolated for the analysis of MSCs by two-photon fluorescence microscopy at indicated time points post transplantation. Notably, the number of tdTomato^+^ cells was ~2-times higher than that of GFP^+^ cells between day 4 and day 28 post transplant (Fig. 5b, c), demonstrating that PDGFB-MSCs secretion of the extracellular protein (PDGF-BB) (Fig. 5d) promoted MSC survival and expansion in the BM. This autocrine signaling produced a fixed-location-independent autoregulatory niche and may yield its own niches to promote MSC proliferation in BM.

Moreover, we sorted the GFP^+^ and tdTomato^+^ cells for single cell RNA-seq using smart-Seq2 protocol at 1 week post transplantation (Fig. 5a). RNA-seq analysis showed 47 upregulated and 282 downregulated genes in PDGFB-tdTomato-MSCs compared to GFP-MSCs (Fig. S5). The dramatically upregulated genes in Gene Ontology (GO) terms were associated with MSC survival and proliferation in vivo such as activation of PDGF signal, response to growth factor and positive regulation of telomere maintenance in PDGFB-tdTomato-MSCs (Fig. 5e). In addition, positive regulation of stem cell proliferation and Wnt signaling pathway were also enriched in PDGFB-tdTomato-MSCs. Activation of the Wnt signaling pathway facilitates the entry of human HSCs into the expanded state, promotes the expansion and reconstitution of HSCs in vivo. It plays an important role in self-renewal and expansion of HSCs [35, 36]. Taken together, these data demonstrate that cotransplanted human MSCs are able to integrate into the functional components of the hematopoietic microenvironment. At the early stage of hematopoietic cell engraftment, PDGFB-MSCs survived better and expanded more robustly than GFP-MSCs. And the secretion of engineering niche factor further supports human HSC engraftment.

## Discussion

High-dose chemotherapy or radiation treatment for hematological malignancies before transplantation damages BM niche cells and reduce the secretion of niche factors, thereby slowing hematopoietic reconstitution because the host microenvironment is unable to provide sufficient support for human hematopoiesis. As such, stem cell niches are an important factor limiting the efficient hematopoietic reconstitution. Accordingly, a simple and effective approach to enhance HSC engraftment is to transplant niche cells and/or factors that can boost niche reconstitution. Multiple factors, such as EGF, FGF2, and PDGFB, have beneficial effects on reconstituting the mouse niche. However, their effects on humanized niches and promoting human HSC engraftment have not been reported. In our current study, after comparing three factors, we have identified PDGFB as a powerful cell factor that increases MSC survival and proliferation after intra-bone injection. The better survival and proliferation of PDGFB-MSCs at the first week after irradiation and transplantation contributed to a 2–4-fold increase in human engraftment.

PDGFB is a chemoattractant and potent mitogen for mesenchymal cells that can stimulate the migration and angiogenesis of endothelial progenitor cells and MSCs [37]. PDGFB is also involved in vessel maturation and stabilization [38]. A previous study [39] showed that the injection of PDGF-BB is necessary and sufficient to enable post-TBI endosteal niche expansion and mouse HSC engraftment. However, we did not observe any positive effects on human HSC engraftment by this approach of factor delivery, which may be due to species differences. The high-level local delivery of PDGF-BB by the transplantation of PDGFB-MSCs should have a significant long-lasting effect. Moreover, the stem cell gene-therapy approach may be safer than intravascular injection of PDGF-BB because optimal levels of PDGF-BB in the BM can be achieved without affecting the baseline levels of PDGF-BB in circulation. In addition, the two-photon-based analysis showed that our engineered PDGFB-MSCs can live in the BM for approximately one month after transplantation, indicating that the gene-therapy approach is safe and without tumorigenicity. Furthermore, we found significant differences in the gene expression pattern of PDGFB-MSCs versus GFP-MSCs. The PDGFB-MSC-specific signature consists of upregulated genes that promote cell survival, proliferation and stem cell maintenance. These data indicate that PDGFB-MSCs can proliferate robustly after transplantation and then establish a more supportive niche than GFP-MSCs in immunodeficient mice via the local production of niche hematopoietic factors.

Recently, several xenotransplantation models for improving the engraftment of human hematopoietic cells have been reported, including models for the in situ subcutaneous generation of human BM-MSC-derived ossicles [40] and the transplantation of engineered bioscaffolds designed to resemble human BM niches [41]. These approaches provide a humanized BM microenvironment that supports hematopoiesis more effectively than existing xenotransplantation models. However, these ossicles or bioscaffolds are not applicable to clinical situations. In contrast, our simple strategy will be more clinically relevant. Our strategy has potential for clinical applications for the following reasons: (1) The use of allogeneic MSCs will abrogate the possibility of the long-term survival and proliferation of transplanted PDGFB-MSCs. (2) More MSCs are only observed at 1–4 weeks after transplantation. One month later, only a few residual MSCs and progeny are still present in the stem cell niche, minimizing safety concerns. (3) Here, we used lentiviral vectors to deliver factors to boost the functionality of MSCs as a proof of principle. However, these can be easily replaced by our recently developed high-efficiency HDR knock-in technology that is based on CRISPR-Cas9 and a double cut donor [42]. (4) We used MSCs as a model for human MSCs. Obviously, other clinically cells, such as human endothelium can also be used to achieve similar therapeutic effects. (5) PDGFB can promote the long-term ex vivo expansion of MSCs, which allows for clonal selection. (6) We envision that the combinatorial use of other factors, such as stem cell factor, may further increase the niche-supporting effects of MSCs.

## Supplementary information

Revised Supplemental Methods and Supplemental Figure Legends

Figure S1

Figure S2

Figure S3

Figure S4

Figure S5

Table S1

Table S2

Table S3

## Data Availability

Publicly available softwares were used in this study. Specific programs including Salmon software (version 0.8.2), DESeq2 program, HTSeq (version 0.9.1), clusterProfiler program and GSEA.

## References

[1] Acar M, Kocherlakota KS, Murphy MM, Peyer JG, Oguro H, Inra CN (2015). Deep imaging of bone marrow shows non-dividing stem cells are mainly perisinusoidal. Nature..

[2] Tomita Y, Sachs DH, Sykes M (1994). Myelosuppressive conditioning is required to achieve engraftment of pluripotent stem cells contained in moderate doses of syngeneic bone marrow. Blood..

[3] Bhattacharya D, Czechowicz A, Ooi AG, Rossi DJ, Bryder D, Weissman IL (2009). Niche recycling through division-independent egress of hematopoietic stem cells. J Exp Med.

[4] Hooper AT, Butler JM, Nolan DJ, Kranz A, Iida K, Kobayashi M (2009). Engraftment and reconstitution of hematopoiesis is dependent on VEGFR2-mediated regeneration of sinusoidal endothelial cells. Cell Stem Cell.

[5] Kopp HG, Avecilla ST, Hooper AT, Shmelkov SV, Ramos CA, Zhang F (2005). Tie2 activation contributes to hemangiogenic regeneration after myelosuppression. Blood..

[6] Zhou BO, Yu H, Yue R, Zhao Z, Rios JJ, Naveiras O (2017). Bone marrow adipocytes promote the regeneration of stem cells and haematopoiesis by secreting SCF. Nat Cell Biol.

[7] Muguruma Y, Yahata T, Miyatake H, Sato T, Uno T, Itoh J (2006). Reconstitution of the functional human hematopoietic microenvironment derived from human mesenchymal stem cells in the murine bone marrow compartment. Blood..

[8] Mendelson A, Frenette PS (2014). Hematopoietic stem cell niche maintenance during homeostasis and regeneration. Nat Med..

[9] Doan PL, Himburg HA, Helms K, Russell JL, Fixsen E, Quarmyne M (2013). Epidermal growth factor regulates hematopoietic regeneration after radiation injury. Nat Med..

[10] Itkin T, Ludin A, Gradus B, Gur-Cohen S, Kalinkovich A, Schajnovitz A (2012). FGF-2 expands murine hematopoietic stem and progenitor cells via proliferation of stromal cells, c-Kit activation, and CXCL12 down-regulation. Blood..

[11] Chen W, Baylink DJ, Brier-Jones J, Neises A, Kiroyan JB, Rundle CH (2015). PDGFB-based stem cell gene therapy increases bone strength in the mouse. Proc Natl Acad Sci USA.

[12] Hu L, Cheng H, Gao Y, Shi M, Liu Y, Hu Z (2014). Antioxidant N-acetyl-L-cysteine increases engraftment of human hematopoietic stem cells in immune-deficient mice. Blood..

[13] Love MI, Huber W, Anders S (2014). Moderated estimation of fold change and dispersion for RNA-seq data with DESeq2. Genome Biol..

[14] Yu G, Wang LG, Han Y, He QY (2012). clusterProfiler: an R package for comparing biological themes among gene clusters. OMICS..

[15] Subramanian A, Tamayo P, Mootha VK, Mukherjee S, Ebert BL, Gillette MA (2005). Gene set enrichment analysis: a knowledge-based approach for interpreting genome-wide expression profiles. Proc Natl Acad Sci USA.

[16] Meng X, Su R-J, Baylink DJ, Neises A, Kiroyan JB, Lee WY-W (2013). Rapid and efficient reprogramming of human fetal and adult blood CD34+ cells into mesenchymal stem cells with a single factor. Cell Res..

[17] in ‘t Anker PS, Noort WA, Kruisselbrink AB, Scherjon SA, Beekhuizen W, Willemze R (2003). Nonexpanded primary lung and bone marrow-derived mesenchymal cells promote the engraftment of umbilical cord blood-derived CD34(+) cells in NOD/SCID mice. Exp Hematol..

[18] Noort WA, Kruisselbrink AB, Anker PSit, Kruger M, Bezooijen RLv, Paus RAd (2002). Mesenchymal stem cells promote engraftment of human umbilical cord blood–derived CD34 cells in NOD/SCID mice. Exp Hematol..

[19] Carrancio S, Romo C, Ramos T, Lopez-Holgado N, Muntion S, Prins HJ (2013). Effects of MSC coadministration and route of delivery on cord blood hematopoietic stem cell engraftment. Cell Transplant..

[20] Kim DH, Yoo KH, Yim YS, Choi J, Lee SH, Jung HL (2006). Cotransplanted bone marrow derived mesenchymal stem cells (MSC) enhanced engraftment of hematopoietic stem cells in a MSC-dose dependent manner in NOD/SCID mice. J Korean Med Sci.

[21] Fernández-García M, Yañez RM, Sánchez-Domínguez R, Hernando-Rodriguez M, Peces-Barba M, Herrera G, et al. Mesenchymal stromal cells enhance the engraftment of hematopoietic stem cells in an autologous mouse transplantation model. Stem Cell Res Ther. 2015;6:165. 10.1186/s13287-015-0155-5 PMC4562358 26345192

[22] Wang Q, Yang Q, Wang Z, Tong H, Ma L, Zhang Y (2016). Comparative analysis of human mesenchymal stem cells from fetal-bone marrow, adipose tissue, and Warton's jelly as sources of cell immunomodulatory therapy. Hum Vaccin Immunother.

[23] Klein C, Strobel J, Zingsem J, Richter RH, Goecke TW, Beckmann MW (2013). Ex vivo expansion of hematopoietic stem- and progenitor cells from cord blood in coculture with mesenchymal stroma cells from amnion, chorion, Wharton's jelly, amniotic fluid, cord blood, and bone marrow. Tissue Eng Part A.

[24] Hiwase SD, Dyson PG, To LB, Lewis ID (2009). Cotransplantation of placental mesenchymal stromal cells enhances single and double cord blood engraftment in nonobese diabetic/severe combined immune deficient mice. Stem Cells..

[25] McKenzie JL, Gan OI, Doedens M, Dick JE (2005). Human short-term repopulating stem cells are efficiently detected following intrafemoral transplantation into NOD/SCID recipients depleted of CD122+ cells. Blood..

[26] Mazurier F, Doedens M, Gan OI, Dick JE (2003). Rapid myeloerythroid repopulation after intrafemoral transplantation of NOD-SCID mice reveals a new class of human stem cells. Nat Med..

[27] Notta F, Doulatov S, Dick JE (2010). Engraftment of human hematopoietic stem cells is more efficient in female NOD/SCID/IL-2Rgc-null recipients. Blood..

[28] Notta F, Doulatov S, Laurenti E, Poeppl A, Jurisica I, Dick JE (2011). Isolation of single human hematopoietic stem cells capable of long-term multilineage engraftment. Science..

[29] Song L, Webb NE, Song Y, Tuan RS (2006). Identification and functional analysis of candidate genes regulating mesenchymal stem cell self-renewal and multipotency. Stem Cells..

[30] Omatsu Y, Seike M, Sugiyama T, Kume T, Nagasawa T (2014). Foxc1 is a critical regulator of haematopoietic stem/progenitor cell niche formation. Nature..

[31] Galun E, Rose-John S (2013). The regenerative activity of interleukin-6. Methods Mol Biol.

[32] Castela M, Nassar D, Sbeih M, Jachiet M, Wang Z, Aractingi S (2017). Ccl2/Ccr2 signalling recruits a distinct fetal microchimeric population that rescues delayed maternal wound healing. Nat Commun..

[33] Sugiyama T, Kohara H, Noda M, Nagasawa T (2006). Maintenance of the hematopoietic stem cell pool by CXCL12-CXCR4 chemokine signaling in bone marrow stromal cell niches. Immunity..

[34] Chou S, Lodish HF (2010). Fetal liver hepatic progenitors are supportive stromal cells for hematopoietic stem cells. Proc Natl Acad Sci USA.

[35] Huang J, Nguyen-McCarty M, Hexner EO, Danet-Desnoyers G, Klein PS (2012). Maintenance of hematopoietic stem cells through regulation of Wnt and mTOR pathways. Nat Med..

[36] Reya T, Duncan AW, Ailles L, Domen J, Scherer DC, Willert K (2003). A role for Wnt signalling in self-renewal of haematopoietic stem cells. Nature..

[37] Fiedler J, Etzel N, Brenner RE (2004). To go or not to go: migration of human mesenchymal progenitor cells stimulated by isoforms of PDGF. J Cell Biochem.

[38] Santo VE, Gomes ME, Mano JF, Reis RL (2013). Controlled release strategies for bone, cartilage, and osteochondral engineering-Part I: recapitulation of native tissue healing and variables for the design of delivery systems. Tissue Eng Part B Rev.

[39] Olson TS, Caselli A, Otsuru S, Hofmann TJ, Williams R, Paolucci P (2013). Megakaryocytes promote murine osteoblastic HSC niche expansion and stem cell engraftment after radioablative conditioning. Blood..

[40] Reinisch A, Thomas D, Corces MR, Zhang X, Gratzinger D, Hong WJ (2016). A humanized bone marrow ossicle xenotransplantation model enables improved engraftment of healthy and leukemic human hematopoietic cells. Nat Med..

[41] Groen RW, Noort WA, Raymakers RA, Prins HJ, Aalders L, Hofhuis FM (2012). Reconstructing the human hematopoietic niche in immunodeficient mice: opportunities for studying primary multiple myeloma. Blood..

[42] Zhang JP, Li XL, Li GH, Chen W, Arakaki C, Botimer GD (2017). Efficient precise knockin with a double cut HDR donor after CRISPR/Cas9-mediated double-stranded DNA cleavage. Genome Biol..

