# Correction: PDGFB-expressing mesenchymal stem cells improve human hematopoietic stem cell engraftment in immunodeficient mice

**DOI:** 10.1038/s41409-020-0781-0

**Published:** 2020-01-10

**Authors:** Xiuxiu Yin, Linping Hu, Yawen Zhang, Caiying Zhu, Hui Cheng, Xiaowei Xie, Ming Shi, Ping Zhu, Xueying Zhao, Wanqiu Chen, Lu Zhang, Cameron Arakaki, Sha Hao, Mei Wang, Wenbin Cao, Shihui Ma, Xiao-Bing Zhang, Tao Cheng

**Affiliations:** 1 State Key Laboratory of Experimental Hematology, National Clinical Research Center for Blood Diseases, Institute of Hematology & Blood Diseases Hospital, Chinese Academy of Medical Sciences & Peking Union Medical College, Tianjin, 300020 China; 2 Department of Medicine, Loma Linda University, 11234 Anderson Street, MC1528B Loma Linda, CA USA; 3 Center for Stem Cell Medicine, Department of Stem Cell & Regenerative Medicine, Chinese Academy of Medical Sciences & Peking Union Medical College, Tianjin, 300020 China

**Keywords:** Haematopoietic stem cells, Bone marrow transplantation

**Correction to: Bone Marrow Transplantation**

10.1038/s41409-019-0766-z

Following the publication of this article incorrect labelling of the axis in Fig. 1c was noted. The *y*-axis title in this figure should read “% CD34” instead of “% CD45”.

**Figure 1:**
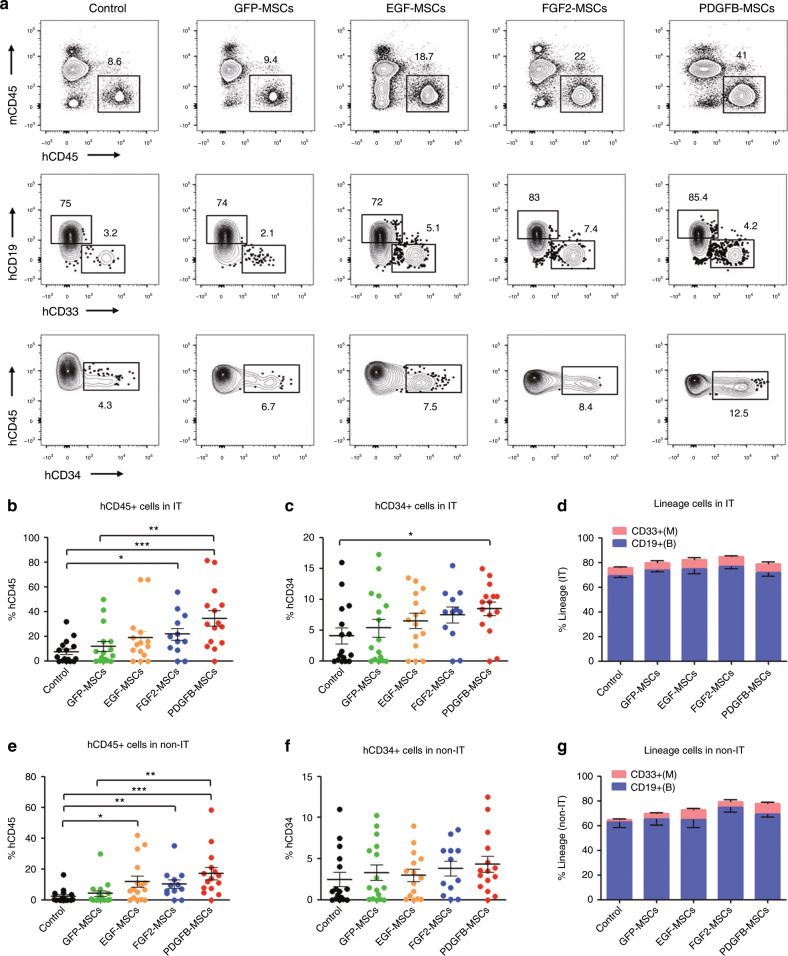
Fig. 1

