# Supplementary material for: PDGFB-expressing mesenchymal stem cells improve human hematopoietic stem cell engraftment in immunodeficient mice
Source: Bone Marrow Transplant. 2019 Dec 5;55(6):1029–40. doi: 10.1038/s41409-019-0766-z (PMC7269905; doi:10.1038/s41409-019-0766-z)
Supplement: Supplementary file 1 — Revised Supplemental Methods and Supplemental Figure Legends [file 41409_2019_766_MOESM1_ESM.docx]

**Supplemental Figure Legends**

**Figure S1. Overexpression of PDGFB promotes MSC proliferation in culture.**

(A) Schematic representation of the lentiviral vectors. ΔRepresents the self-inactivation design by partially deleting the U3 domain of the 3’ long terminal repeat. cPPT, central polypurine tract; RRE, rev-responsive element; Wpre, posttranscriptional regulatory element; ψ, packaging signal. (B) mRNA and (C) protein levels of EGF, FGF2, PDGFB in GFP-MSCs, EGF-MSCs, FGF2-MSCs, PDGFB-MSCs, individually. The mRNA expression level of each gene in GFP-MSCs (control) was normalized to 1 and the mRNA expression level in other cells was shown relative to that in GFP-MSCs (The primers used in this study were shown in the Table S2). (D) Proliferation curve of GFP-MSCs, EGF-MSCs, FGF2-MSCs and PDGFB-MSCs in in vitro culture. *p < 0.05; **p =0.01 to 0.001; ***p < 0.001.

**Figure S2.** **Human cell engraftment in** **the NOD-SCID mice 12-weeks post transplantation.**

(A) Human CB-CD34^+^ cells were co-transplanted with 1×10^6^ GFP-MSCs into the right tibia of NOD-SCID mice. Only CB-CD34^+^ cells were injected in the control group. Mean level of human CD45^+^ cells in the IT, BM and SP of the mice. (B-D) Human CB-CD34^+^ cells were mixed with 5×10^5^ GFP-MSCs, EGF-MSCs, FGF2-MSCs or PDGFB-MSCs and injected into the right tibia of NOD-SCID mice. In the control group, only CB-CD34^+^ cells were injected. (B) Mean level of human CD45^+^ cells in SP. (C) Lineage potential of human cells in SP. (D) The percentage of human CD45^+^CD19^+^IgM^+^ B cells in SP. n=12 to 16 per group, *p < 0.05; **p =0.01 to 0.001.

**Figure S3. Hematopoietic cell engraftment and lineage development in the spleen in NOG mice.**

(A) Representative flow cytometric analysis of engraftment and lineage differentiation in the spleen of NOG mice after 16 weeks of transplant. (B) The level of human CD45^+^ cells in the spleen of NOG mice. (C) Lineage engraftment (CD19^+^ B cells, CD3^+^ T cells, CD56^+^ NK cells) in the spleen. n=8 to 17 per group, 3 independent experiments; *p < 0.05; **p =0.01 to 0.001; ***p < 0.001.

**Figure S4. PDGFB-MSCs support the self-renewal of human HSCs in NOG mice.**

(A) Mean engraftment of human cells (CD45^+^) from BM of secondary recipients. (B) Lineage engraftment expressed as frequency of human CD45^+^ cells. n=4 to 9 per group, *p < 0.05.

**Figure S5. Gene expression of PDGFB-tdTomato-MSCs and GFP-MSCs in vivo.** Scatter plot of global mRNA profiling showing upregulated (red) and down-regulated (blue) genes in PDGFB-tdTomato-MSCs versus GFP-MSCs after injected into NOD-SCID mice for 1 week (Selection criteria: p ≤ 0.01, ≥ 2-fold difference).

**Table S3. Limiting dose analysis of CD34^+^ CB cells in NOD-SCID mice after cotransplanted with GFP-MSCs and PDGFB-MSCs.** Six- to eight-week-old irradiated mice were injected in the right tibia at limiting doses with CD34^+^ CB cells (2 500, 5 000, 10 000, or 20 000). Human cell engraftment (hCD45^+^) was measured 12-13 weeks after injection. Using the number of positively engrafted mice at each dose, the frequency of SRC was calculated using L-Calc software. The data show the mean frequency (± 95% confidence interval).

**Supplemental Methods**

**Cell culture and** **lentiviral transduction**

MSCs used in this study are induced mesenchymal stem cells from human cord blood CD34+ cells by direct reprogramming with episomal vector-mediated transient OCT4 expression, as described in our previous report. MSCs were cultured in medium consisted of α-MEM (Life Technologies; 32571-036), 2% fetal bovine serum (FBS, ABM; TM999-500), 5% serum replacement (Invitrogen; 10828-028), PDGF-BB (20 ng/ml, ABM; Z100355), EGF (20 ng/ml, ABM; Z200025), FGF2 (20 ng/ml, ABM; Z101455), 0.1% ITS (Life Technologies; 41400-045), 0.1% AAP (Sigma-Aldrich; 49752) and 1% penicillin/streptomycin (ABM, G255). Cells were cultured in non-TC culture plates precoated with human fibronectin (BD), and the culture plates were placed in a hypoxia chamber that was flushed with mixed air containing 92% N_2_, 5% CO_2_ and 3% O_2_. The cells were changed medium 3 times a week and passaged every 3-5 days after treatment with accutase for 5 minutes. MSCs at passages 3-6 were transduced with Lenti EF1-GFP, Lenti EF1-EGF, Lenti EF1-FGF2, Lenti EF1-PDGFB, or Lenti EF1-tdTomato at a multiplicity of infection of 1-2 for 6 hours. After transduction, cells were cultured in a hypoxia chamber and passaged every 2-3 days. All the cells were verified to be mycoplasma negative before undertaking any experiments with them.

**MSC proliferation assay**

For the MSC proliferation assay, 1 × 10^5^ cells were plated in each well of multiple six well plates. The culture medium was changed every 2 days. The cell number was determined every 3 days.

**Mice**

Mice used in our study included female nonobese diabetic-severe combined immunodeficiency (NOD-SCID) and NOD/Shi-scid/IL2Rg^null^ (NOG) mice at 6 to 8 weeks of age, purchased from the Institute of Laboratory Animals, Chinese Academy of Medical Sciences (Beijing, China), and housed in a specific-pathogen-free facility. Experimental protocols were approved by the Institute of Hematology Animal Care and Use Committee. To ensure the accuracy of the study, each experiment contains at least 4 randomly assigned mice. Mice were irradiated at a dose of 250 cGy 24 hours prior to transplantation. The NOD-SCID mice were anesthetized and cotransplanted with CD34^+^ cells and MSCs through the right tibia by a 28-gauge needle. For NOG mice, we injected MSCs in both tibias and then transplanted human CD34^+^ cells intravenously. At 12 weeks (for NOD-SCID) or 16 weeks (for NOG) after transplantation, the mice were sacrificed. For bone tissue imaging assay, mice were irradiated at a dose of 250 cGy 24 hours before experiment. Then, we transplanted 5x10^5^ GFP-MSCs or PDGFB-tdTomato-MSCs into their tibias. The tibias of the transplanted mice were isolated and analyzed by two-photon fluorescence microscopy at indicated time points post-transplantation. The data presented did not require the use of blinding.

**Western blots**

Cells were collected and washed with PBS. Two million cells were lysed with 100 μl of 2×SDS sample buffer, sonicated for 5 min and then boiled at 95°C for 5 min. The extractions were centrifuged at 12 000 rpm at 4°C for 10 min, resolved by SDS-PAGE and electrophoretically transferred to polyvinylidene difluoride membranes (Millipore). The primary antibodies used included anti-EGF (Abcam, 1:1 000; ab9695), anti-FGF2 (Abcam, 1:250; ab16828), anti-PDGFB (Abcam, 1:1 000; ab23914) and anti-β-actin (Cell Signaling Technology, 1:1 000; 3700). Horseradish peroxidase–conjugated antibodies to rabbit (Cell Signaling Technology, 1:4 000; 7074) and mouse (Cell Signaling Technology, 1:2 000; 7076) IgG were used as the secondary antibodies. Western blots were visualized using ECL detection reagents (Millipore) and detected with the Luminescent Image Analyzer (ImageQuant LAS 4000).

**Bone tissue imaging**

Freshly dissected tibias were fixed overnight in the cold (4°C) 4% paraformaldehyde (PFA) while shaking. Wash the bones with PBS to remove the PFA, then the bones were embedded in chilled SCEM embedding media within a tissue-tek cryomold. The tissue-tek cryomolds, with the embedded bones, were submerged in hexane in dry ice and stored at -80°C. A Leica cryostat was used to bisect the bones longitudinally. Intact half bone was washed with PBS to remove SCEM and then subjected to nuclear staining with DAPI. All staining processes were performed at room temperature in Eppendorf tubes on a rotator. Fluorescence images were acquired with an Olympus two-photon microscope and confocal microscope (FV1200MPE). The bone was imaged by second harmonic generation (SHG) with 920 nm excitation, with 488-nm and 559-nm visible laser lines for GFP and tdTomato fluorescence, respectively. We used Olympus software (FV10-ASW) for image processing.

**Single-cell RNA sequencing and data analysis**

Single-cell libraries for RNA sequencing were prepared using a modified smart-seq2 protocol. Briefly, single cells were sorted into 0.2 mL thin-wall 8 strip PCR tubes containing 2.55 µl cell lysis solution. The reverse transcription primers were anchored with cell-specific barcode and the unique molecular identifier (UMI). The cDNAs were amplified by 20 cycles of PCR, and PCR products were pooled together for purification and library construction. Further 4 cycles of PCR were used to amplify the cDNAs and introduce the biotin tags to the 3’ ends of the amplified PCR products. A 150-bp paired-end sequencing was performed on a HiSeq4000 (Illumina) (sequenced by Novogene). The raw reads of Smart-seq2 were trimmed [1]. Subsequently, the clean reads were aligned to the hg38 human genome using Hisat2 [2]. Processed reads were then counted by HTSeq [3] (version 0.9.1). Differentially expressed genes (DEGs) were calculated using DESeq2 [4] with the cutoff p value of less than 0.01 and absolute fold change of more than 2. Gene Ontology (GO) and KEGG pathway enrichment analysis based on DEGs were carried out by enrichR [5].

**REFERENCES**

1. Li L, Dong J, Yan L, Yong J, Liu X, Hu Y, et al. Single-Cell RNA-Seq Analysis Maps Development of Human Germline Cells and Gonadal Niche Interactions. Cell Stem Cell. 2017;20:891-892.

2. Kim D, Langmead B, Salzberg SL. HISAT: a fast spliced aligner with low memory requirements. Nat Methods. 2015;12:357-360.

3. Anders S, Pyl PT, Huber W. HTSeq--a Python framework to work with high-throughput sequencing data. Bioinformatics. 2015;31:166-169.

4. Love MI, Huber W, Anders S. Moderated estimation of fold change and dispersion for RNA-seq data with DESeq2. Genome Biol. 2014;15:550.

5. Chen EY, Tan CM, Kou Y, Duan Q, Wang Z, Meirelles GV, et al. Enrichr: interactive and collaborative HTML5 gene list enrichment analysis tool. BMC Bioinformatics. 2013;14:128.
