# Supplementary figures and images for: PDGFB-expressing mesenchymal stem cells improve human hematopoietic stem cell engraftment in immunodeficient mice
Source: Bone Marrow Transplant. 2019 Dec 5;55(6):1029–40. doi: 10.1038/s41409-019-0766-z (PMC7269905; doi:10.1038/s41409-019-0766-z)

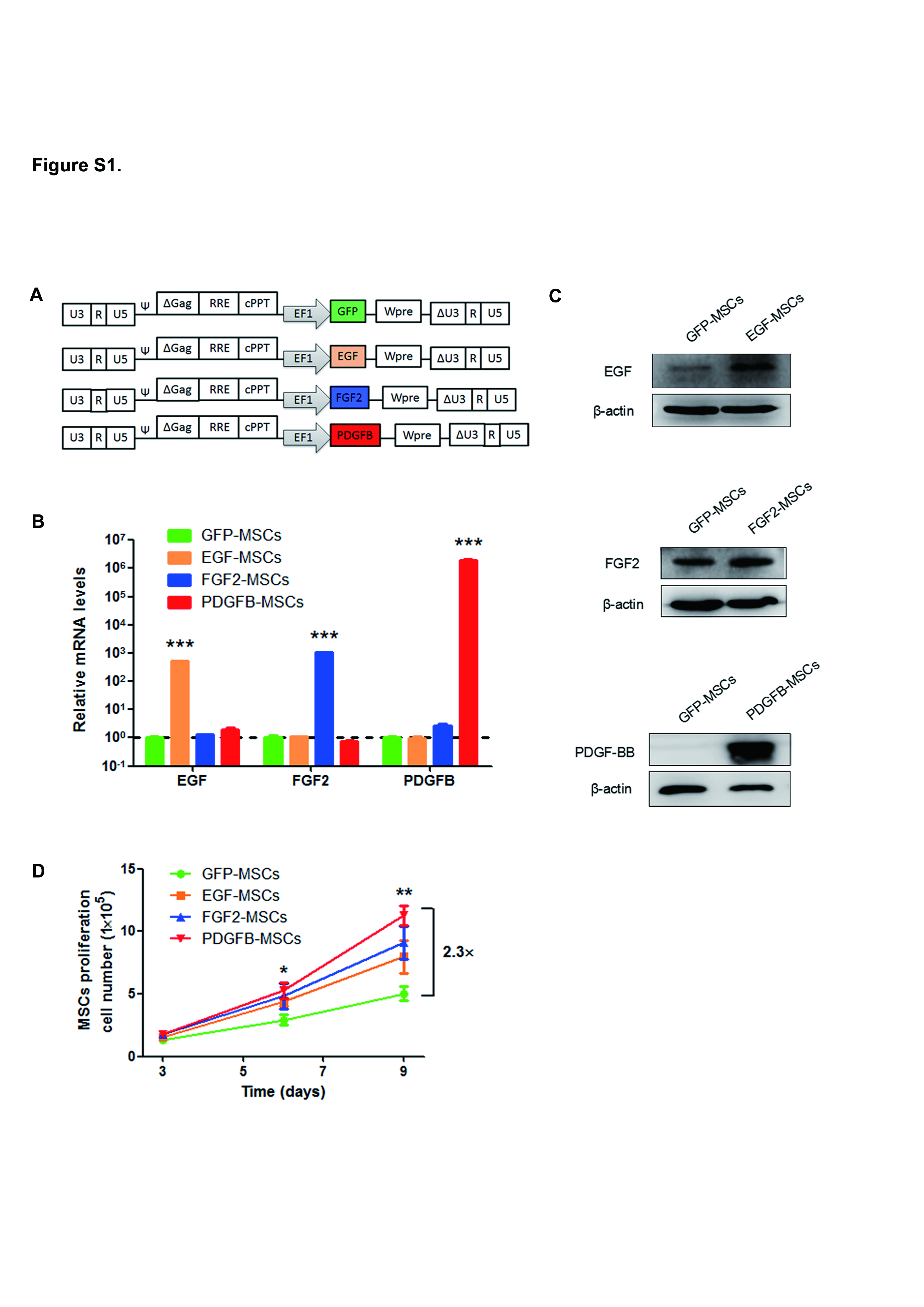

Supplement: Supplementary file 2 — Figure S1 [file 41409_2019_766_MOESM2_ESM.tif]

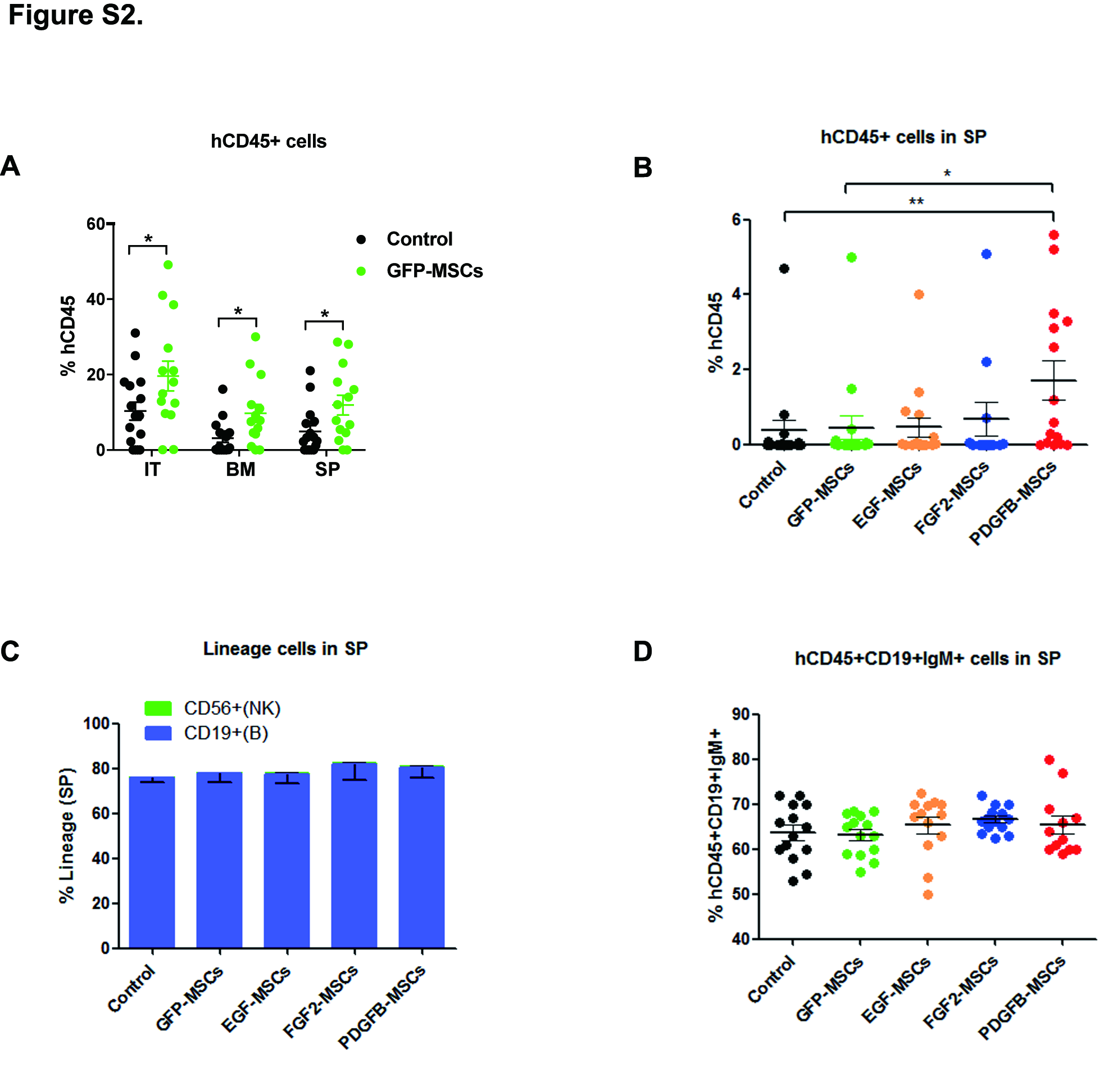

Supplement: Supplementary file 3 — Figure S2 [file 41409_2019_766_MOESM3_ESM.tif]

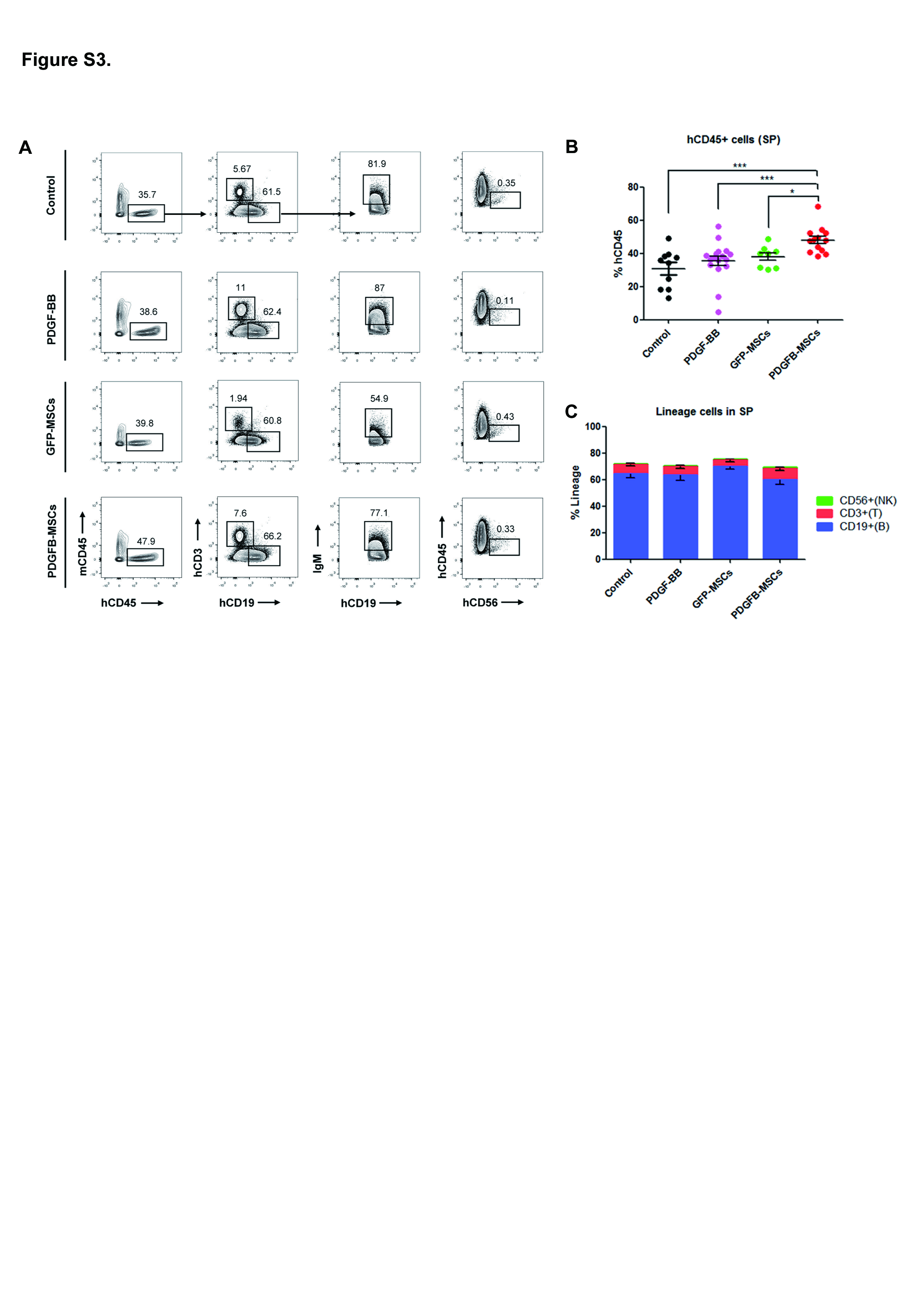

Supplement: Supplementary file 4 — Figure S3 [file 41409_2019_766_MOESM4_ESM.tif]

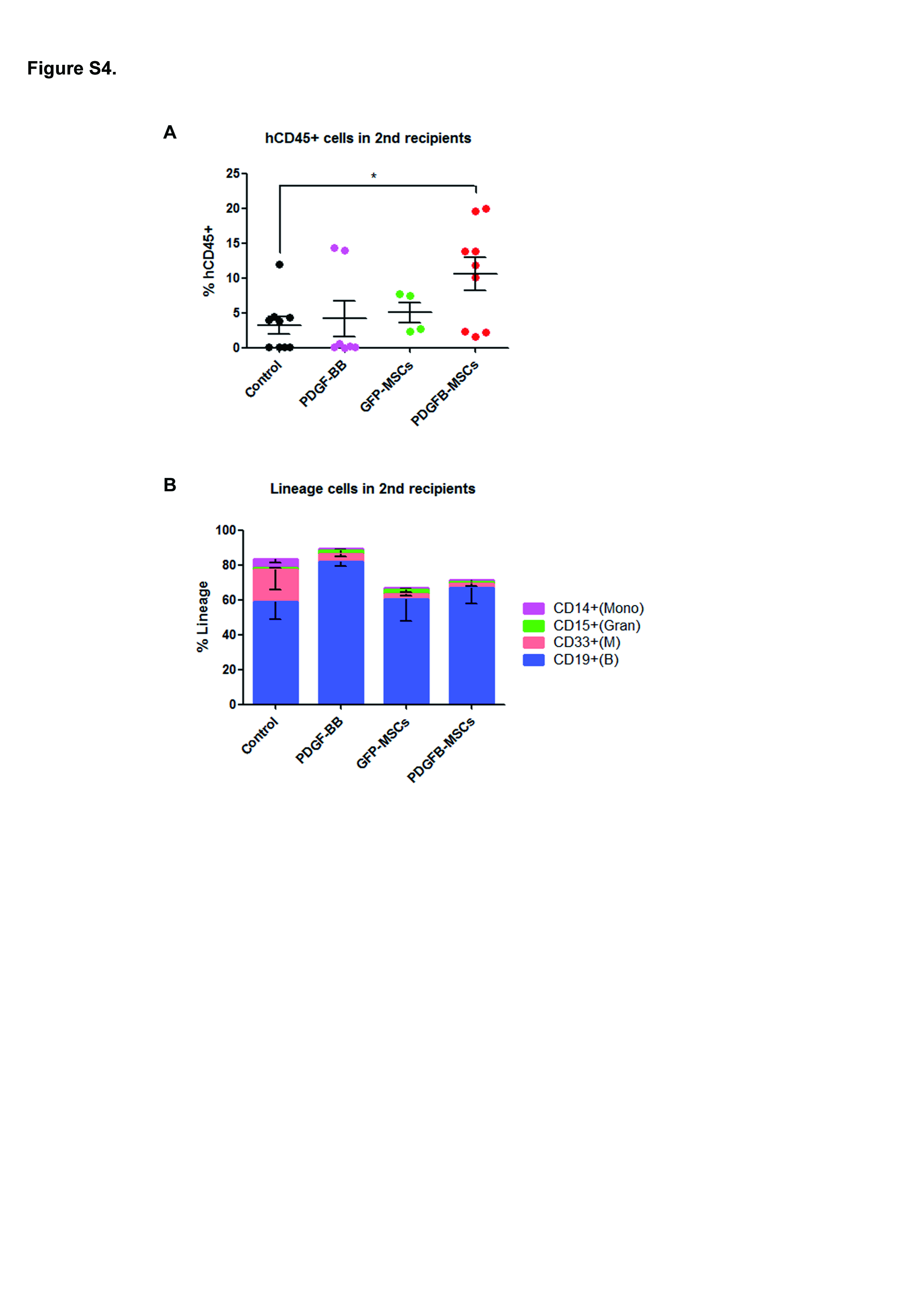

Supplement: Supplementary file 5 — Figure S4 [file 41409_2019_766_MOESM5_ESM.tif]

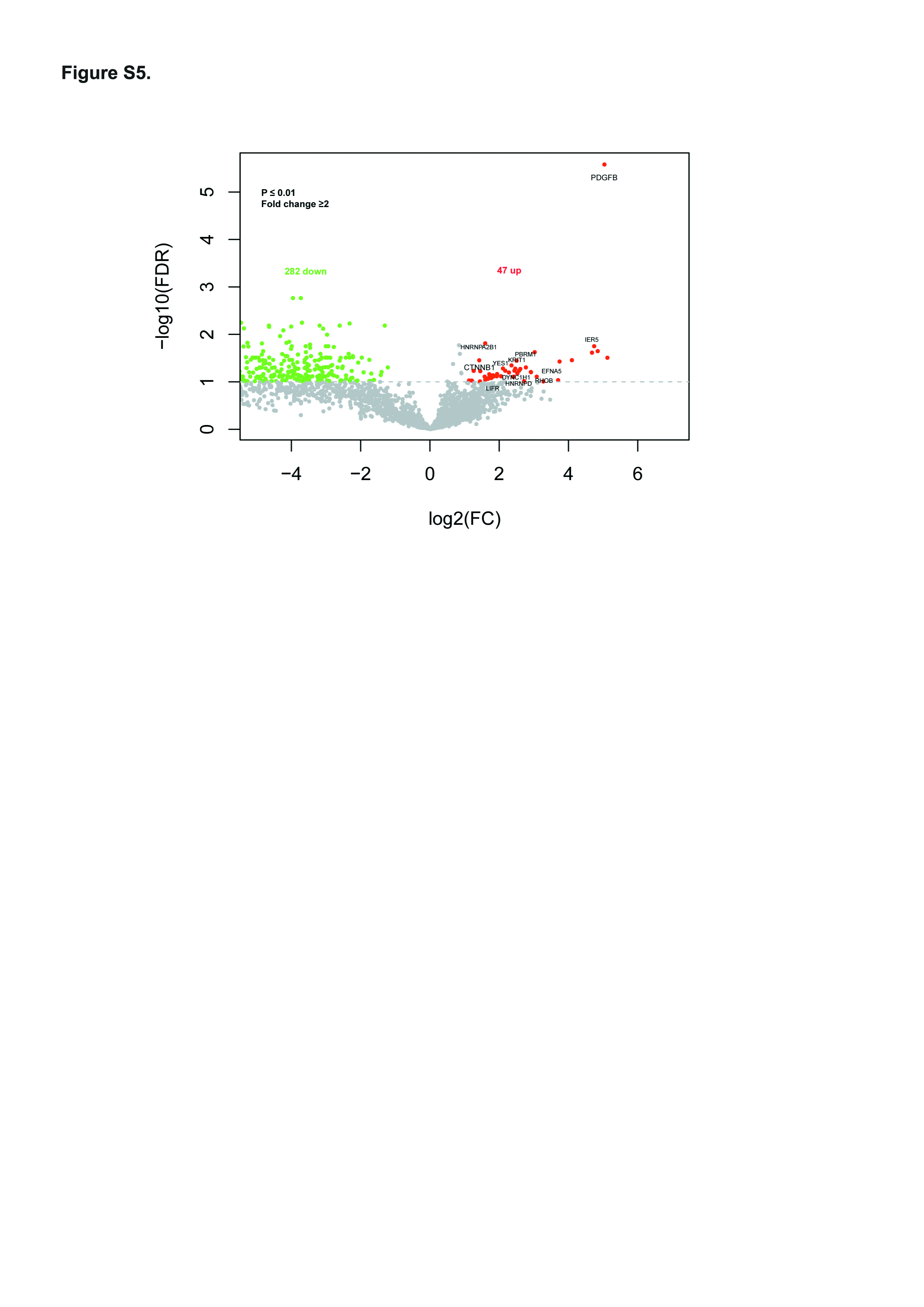

Supplement: Supplementary file 6 — Figure S5 [file 41409_2019_766_MOESM6_ESM.tif]
