# Supplementary material for: PDGFB-expressing mesenchymal stem cells improve human hematopoietic stem cell engraftment in immunodeficient mice
Source: Bone Marrow Transplant. 2019 Dec 5;55(6):1029–40. doi: 10.1038/s41409-019-0766-z (PMC7269905; doi:10.1038/s41409-019-0766-z)
Supplement: Supplementary file 7 — Table S1 [file 41409_2019_766_MOESM7_ESM.docx]

**Table S1. Antibodies for flow cytometry used in this study**

| Markers | Brand/Cat No. | |
| --- | --- | --- |
| anti-human CD45 | PE-Cy | BD/557748 |
|  | APC-Cy7 | BD/348795 |
|  | PE | BD/555483 |
| anti-human CD34 | APC | BD/555824 |
| anti-human CD38 | PE-Cy7 | BD/335790 |
| anti-human CD45RA | FITC | BD/555488 |
| anti-human CD90 | Percp-Cy5.5 | BD/561557 |
| anti-human CD49f | BV605 | BD/740416 |
| anti-human CD33 | APC | BD/340474 |
| anti-human CD19 | PE | BD/349209 |
| anti-human CD3 | FITC | BD/555332 |
| anti-human CD235a | PE | Coulter/IM2211U |
| anti-human CD56 | Percp-Cy5.5 | BD/560842 |
| anti-human IgM | APC | BD/551062 |
| anti-human CD4 | APC | BD/565994 |
| anti-human CD8 | PE | BD/555635 |
| anti-human CD15 | V450 | BD/642917 |
| anti-human CD14 | PE-CY7 | Coulter/A22331 |
| anti-mouse CD45 | Percp-Cy5.5 | BD/550994 |
|  | APC-Cy7 | BD/557659 |
