# Supplementary material for: PDGFB-expressing mesenchymal stem cells improve human hematopoietic stem cell engraftment in immunodeficient mice
Source: Bone Marrow Transplant. 2019 Dec 5;55(6):1029–40. doi: 10.1038/s41409-019-0766-z (PMC7269905; doi:10.1038/s41409-019-0766-z)
Supplement: Supplementary file 8 — Table S2 [file 41409_2019_766_MOESM8_ESM.docx]

**Table S2. Primers used in this study**

| Genes | Primer sequences | |
| --- | --- | --- |
| GAPDH | Forward primer | AGAAGACTGTGGATGGCCCCTC |
| GAPDH | Reverse primer | GATGACCTTGCCCACAGCCTT |
| EGF | Forward primer | TGTCCACGCAATGTGTCTGAA |
| EGF | Reverse primer | CATTATCGGGTGAGGAACAACC |
| FGF2 | Forward primer | AGTGTGTGCTAACCGTTACCT |
| FGF2 | Reverse primer | ACTGCCCAGTTCGTTTCAGTG |
| PDGFB | Forward primer | CTTTAAGAAGGCCACGGTGA |
| PDGFB | Reverse primer | CGAATGGTCACCCGAGTT |
| PDGFRB | Forward primer | GGAGAGGGCAGTAAGGAGGA |
| PDGFRB | Reverse primer | TCGCCTTGCTGCTGATGG |
| PDGFA | Forward primer | AAGCAGCCAACCAGATGTGA |
| PDGFA | Reverse primer | GGAGGAGAACAAAGACCGCA |
| FZD7 | Forward primer | TGAACAAGTTCGGCTTCCAGT |
| FZD7 | Reverse primer | TAGGGCGCGGTAGGGTAG |
| AXL | Forward primer | GCACTTACAAGACTTGGTCCC |
| AXL | Reverse primer | AGAAAGAGGATGTCTTGTTCAGC |
| NT5E | Forward primer | TGCAACATGGGCAACCTGAT |
| NT5E | Reverse primer | TGGATTCCATTGTTGCGTTCA |
| RBM24 | Forward primer | TTAAGCGCGCTGTTGCTTTG |
| RBM24 | Reverse primer | AGCCATGGTGACCTTTGCAG |
| UGCG | Forward primer | GTGGCAGCAGCTTTGCTTTA |
| UGCG | Reverse primer | CCTCGCCATGTGTTGTTCAC |
| CRIM1 | Forward primer | CTGAGTCCTGGAAGCCTGAC |
| CRIM1 | Reverse primer | GGACATACATTTCTATGCAGTAGGG |
| HSPG2 | Forward primer | AGCATCTCAGGAGACGACCT |
| HSPG2 | Reverse primer | GAAATTCACCAGGGCTCGGA |
| MN1 | Forward primer | ACCTCGGCCAGTAAATTGGG |
| MN1 | Reverse primer | CGATCATGTTCTGGCAAGCG |
| MAP2K3 | Forward primer | TTATGGGCCACAACAGGTCC |
| MAP2K3 | Reverse primer | TCTTGGATTTTCCTGCCCCG |
| CCL2 | Forward primer | TCTGTGCCTGCTGCTCATAG |
| CCL2 | Reverse primer | GGGCATTGATTGCATCTGGC |
| IGF2 | Forward primer | CTCCGTCGGCCCAAACC |
| IGF2 | Reverse primer | CACAGAGAAGCGGAGGGAAG |
| CXCL12 | Forward primer | TGCCCTTCAGATTGTAGCCC |
| CXCL12 | Reverse primer | CTGTAAGGGTTCCTCAGGCG |
| DLK1 | Forward primer | CCTGCAGCACACCCAGG |
| DLK1 | Reverse primer | GGTCTCGCACTTGTTGAGGA |
| ATF3 | Forward primer | AGTTGACCAACCATGCCTTGA |
| ATF3 | Reverse primer | ATTTTGGGGCAAGGTGCTGA |
| IL6 | Forward primer | CTTCGGTCCAGTTGCCTTCT |
| IL6 | Reverse primer | TGGAATCTTCTCCTGGGGGT |
| DCN | Forward primer | GGGTGGGGAAACAAACCCTT |
| DCN | Reverse primer | TGCAGTATAGTGGAGCAACTTGA |
| IBSP | Forward primer | AAGGGCACCTCGAAGACAAC |
| IBSP | Reverse primer | CCCTCGTATTCAACGGTGGT |
| GLUL | Forward primer | GACCAGAACACCTTCCACCA |
| GLUL | Reverse primer | GGACTTTCTCACCCTGAGGC |
| IER3 | Forward primer | GTCTGGTGGTGGGTCGTAAG |
| IER3 | Reverse primer | CGCCGAAGTCTCACACAGTA |
| EDNRB | Forward primer | AAAGCAGAGACGGGAAGTGG |
| EDNRB | Reverse primer | CTGCTGAGGTGAAGGGGAAG |
