# Supplementary material for: PDGFB-expressing mesenchymal stem cells improve human hematopoietic stem cell engraftment in immunodeficient mice
Source: Bone Marrow Transplant. 2019 Dec 5;55(6):1029–40. doi: 10.1038/s41409-019-0766-z (PMC7269905; doi:10.1038/s41409-019-0766-z)
Supplement: Supplementary file 9 — Table S3 [file 41409_2019_766_MOESM9_ESM.docx]

**Table S3. Limiting dose analysis in NOD-SCID mice**

| Tissue | Group | Frequency of SRC | |
| --- | --- | --- | --- |
|  |  | Mean | Range (±95%CI) |
| Injected Tibia | GFP-MSCs | 1/8 908 | 6 276-12 645 |
|  | PDGFB-MSCs | 1/4 353 | 3 092-6 129 |
| Non-injected Tibia | GFP-MSCs | 1/17 949 | 11 903-27 067 |
|  | PDGFB-MSCs | 1/5 302 | 3 775-7 447 |
| Spleen | GFP-MSCs | 1/23 322 | 14 903-36 497 |
|  | PDGFB-MSCs | 1/8 969 | 6 317-12 736 |
